# Comparison of Methods for the Isolation of Salivary Extracellular Vesicles

**DOI:** 10.3390/ijms27114899

**Published:** 2026-05-28

**Authors:** Ulrike Kegler, Anja Buhmann, Heinz-Peter Friedl, Manuela Hofner, Christa Noehammer

**Affiliations:** Competence Unit Molecular Diagnostics, Center for Health & Bioresources, Austrian Institute of Technology GmbH, Giefinggasse 4, 1210 Vienna, Austria

**Keywords:** extracellular vesicles, EV isolation, saliva, DNA methylation, microRNA, non-invasive diagnostics

## Abstract

Extracellular vesicles (EVs) have attracted growing attention for their diagnostic and prognostic potential as they carry molecular cargo such as DNA, RNA, proteins and lipids derived from their cells of origin. While EV research has traditionally focused on blood, this study explicitly explored saliva as a promising, non-invasive sample matrix for EV isolation and biomarker discovery. Six different EV isolation methods were compared for their ability to recover salivary small EVs suitable for downstream DNA and microRNA analysis. Nanoparticle tracking analysis (NTA) revealed variation in vesicle sizes, concentrations and surface charges across all tested EV isolation approaches. In addition to being the fastest and simplest isolation method, the miRCURY Exosome Isolation kit—serum and plasma from Qiagen (ExiQ) also resulted in the highest EV yields with average particle sizes of ~130 nm. Western blot analysis further verified the presence of EV-specific markers (CD9, Alix) and no detectable signal for ApoA1 as an indicator for lipoprotein contamination, underscoring the purity of ExiQ-isolated vesicles. Always applying the same protocol for parallel DNA and RNA isolation on vesicles extracted by various methods, differences in DNA and RNA yields were observed across the evaluated isolation kits. ExiQ-isolated EVs showed the best recovery for both nucleic acid types. Notably, nuclease treatment of isolated EVs revealed that substantial amounts of DNA were present on the EV surface, whereas microRNA was predominantly localized within the vesicles. The present study, extensively comparing different EV isolation methods, demonstrates that salivary EVs are a viable source for non-invasive diagnostics and suggests the miRCURY Exosome Isolation kit—serum and plasma from Qiagen (ExiQ) to be a good choice for integration in future salivary EV-based diagnostic assays given its simplicity, speed and excellent performance.

## 1. Introduction

**Figure 1:**
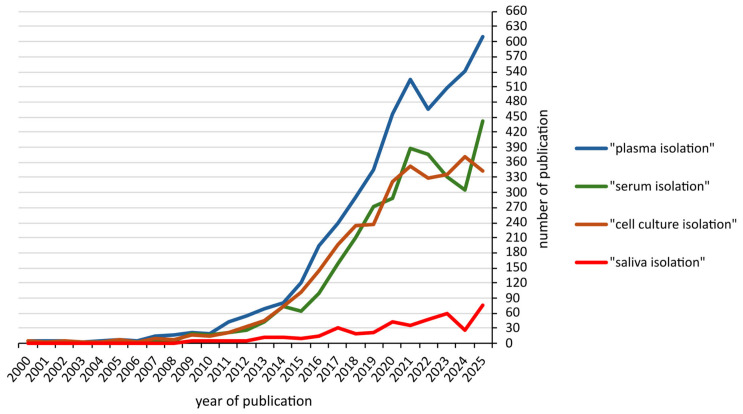
Number of PubMed-listed publications per year related to EV isolation, categorized by biological matrix. The search term “(2025 [Date-Publication]) AND (exosome … isolation)” was used to retrieve publication counts from PubMed and grouped by different biological matrices: plasma, serum, cell culture and saliva. Publications containing either “exosome […]” or “EV […]” were combined. The *x*-axis indicates the publication year, and the y-axis shows the number of publications per year.

An initial idea of exosomes emerged when the biochemists Pan and Johnstone shared their major discovery with the world in 1983, that transferrin receptors are associated with small vesicles during the maturation of reticulocytes, and their formation was visualized using anti-transferrin receptor antibodies [1]. A few years later, the term “exosome” for these vesicles was also coined by Rose Johnstone [2]. Although these findings were impressive, they did not arouse further interest in the scientific world for two decades. The turning point was in the early 2000s when the potential of exosomes was recognized and exosomes came into focus, which is also reflected in the steady year-by-year increase in the number of publications related to exosome isolation (Figure 1). Exosomes are small extracellular vesicles (EVs) surrounded by a membrane-protein-containing lipid bilayer. Since an agreement about specific hallmarks of extracellular vesicle subtypes has not yet been reached, the MISEV 2018 and 2023 guidelines recommend classifying extracellular vesicles either by physical characteristics such as size or density, or by their biological composition [3,4]. With respect to size, EVs can be categorized as small EVs (<200 nm), including exosomes, and large EVs (>200 nm) [3,4]. Exosomes fall into the category of small EVs with a size ranging up to 150 nm [5]. Exosomes are constantly secreted by cells. Predominantly, the biogenesis of exosomes begins with the formation of intraluminal vesicles (ILVs) within multivesicular endosomes (MVEs). In turn, the membrane of the MVEs fuses with the plasma membrane of the cell, resulting in the release of ILVs. In the process of secreting, the ILVs are now called exosomes [6]. Small EVs, including exosomes, can derive from all healthy as well as abnormal cells and are found in all body fluids, such as plasma, serum, urine, amniotic fluid, breast milk, cerebrospinal fluid or saliva [7,8,9]. Numerous studies have shown that EVs can have various functions depending on the type and the condition of the cell of origin and that they are involved in biological processes such as angiogenesis, inflammation, apoptosis, antigen presentation, coagulation and cellular homeostasis [10,11,12,13]. There is emerging evidence that EV-mediated cell-to-cell communication is of importance in both health and disease. The cargo of EVs includes DNA, microRNA, messenger RNA, proteins and lipids [14,15]. Based on these facts, EVs hold tremendous potential to be used for diagnosis and prognosis of diseases and could also open up novel and interesting opportunities for the development of bio-markers [15,16].

This potential for diagnostic use brings about the need to optimize and standardize EV isolation. There already exists substantial work describing several ways to purify small EVs from biological matrices. As shown in Figure 1, most of this research focuses on EV isolation from serum, plasma and cell culture media [17,18,19,20,21,22]. Nevertheless, so far, no gold standard for small EV isolation exists. Numerous commercial kits relying on a broad spectrum of vesicle preparation techniques and principles are available on the market, and new approaches are consistently introduced [23]. Among these methods are, e.g., ultrafiltration, magnetic beads harboring antibodies for EV-specific capture, precipitation or size exclusion chromatography. Ultracentrifugation, often considered as the gold standard, is still the most commonly used approach [24,25].

In contrast to many other studies, this study specifically investigates EV preparation from saliva, a widely unexplored but most interesting sample matrix. Saliva is a biological fluid that is produced by three pairs of major and several minor salivary glands and is secreted into the oral cavity. In addition to 99% water, saliva consists of inorganic salts, enzymes, proteins and nucleic acids [26,27]. Saliva represents a very attractive liquid biopsy for diagnostic applications. The obvious advantage is the non-invasive, painless and easy way of sample collection without the need for skilled personnel. Furthermore, it is almost an unlimited source due to a production of up to 1 L of fluid per day [28,29]. All these characteristics make saliva a suitable and very easy-to-access sample matrix for future non-invasive, EV-based diagnostics, taking advantage of salivary EV-derived biomarkers.

Along these lines, the objective of this study was to test and compare six different strategies for isolating salivary small EVs. For this purpose, saliva from healthy participants was used as starting material and tested whether this body fluid is suitable for non-invasive biomarker development, including, for example, DNA methylation or microRNA biomarkers. Alongside the comparison of EV isolation approaches presented here, not only was the recovery of exosomal DNA and miRNA evaluated, but the isolated EVs were also characterized with respect to their size, concentration, and specific surface and cargo proteins.

## 2. Results

### 2.1. Experimental Setup

**Figure 2:**
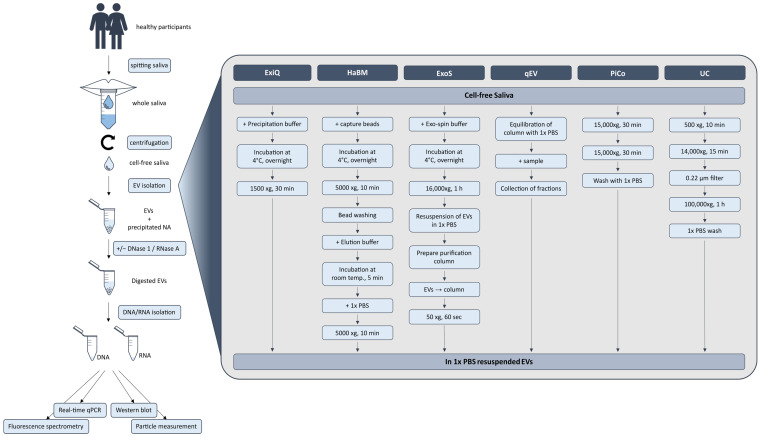
Workflow of the EV isolation approaches comparison. Whole saliva samples were collected from healthy participants by unstimulated drooling. The cell-free saliva was obtained by separation of cell particles from the aqueous part via centrifugation. For the comparison, salivary EVs were purified using 6 different methods: miRCURY Exosome Isolation kit—Serum and Plasma (ExiQ) (Qiagen); immunobeads for overall exosome capture from human biological fluids—plasma, serum, urine (HaBM) (Hansa Bio Med); Exo-Spin Exosome Purification Kit for cell culture media/urine/saliva (ExoS) (Cell Guidance System); qEVoriginal Size Exclusion Column (qEV) (iZON); Pierce Concentrator, PES, 100K MWCO, 0.5 mL (PiCo) (Thermo Fisher Scientific) and differential ultracentrifugation (UC). To get rid of the outer nucleic acids that may have been co-precipitated or are a priori attached to the extracted EVs, samples were treated with DNase 1 and RNase A. The DNA and RNA inside the vesicles were subsequently isolated using a co-purification kit ZR-Duet DNA/RNA MiniPrep which simultaneously extracts DNA and RNA in separate fractions. Finally, downstream analyses including Real-Time qPCR, fluorescence spectrometry, Western blot and particle measurement were performed.

We evaluated six different kits and methods for their feasibility to extract EVs from cell-free saliva (for an overview of the tested kits and the specific EV isolation workflows, see Figure 2). The selection of kits was done based on having a good representation of various underlying preparation principles and also taking into account practicability in clinical settings, with rather limited hands-on times.

As a first step, cells present in saliva and debris from them were precipitated via centrifugation from whole saliva, which had been collected from four healthy probands. The thereby obtained supernatants, representing cell-free saliva, were merged in equal proportions and mixed to obtain a homogenous cell-free saliva pool as starting sample material. In our comparison study, 1 mL of cell-free saliva pool was applied in six replicates to each EV isolation kit. The manufacturer’s instructions were followed. As the next step, three out of the six EV isolation replicates from each kit were treated with DNase 1 and RNase A to determine the actual DNA and RNA amounts present inside the EVs, in contrast to the amounts obtained from the untreated EV resuspensions. Subsequently, a parallel DNA and RNA isolation was performed from each sample, resulting in two separate fractions of DNA and RNA. All isolation samples were eluted in equal volumes (100 µL). All subsequent analyses performed are explained in more detail in the following sections.

### 2.2. Characterization of Isolated EVs via Nanoparticle Tracking Analysis (NTA)

**Figure 3:**
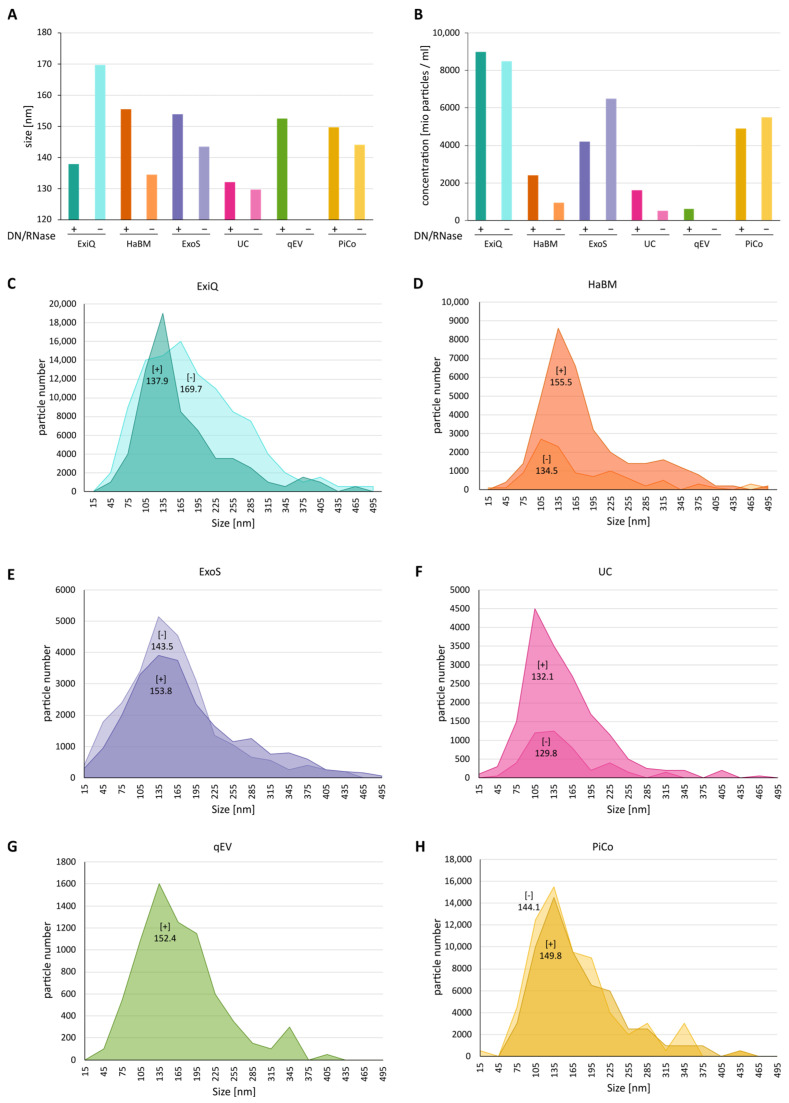
Characteristics of EVs measured by NTA. Particle size (**A**) and amount (**B**) for all 6 EV isolation methods in DN/RNase-treated and untreated samples were analyzed using NTA by ZetaView. The distribution of the particle number over a size range is displayed for the treated and untreated samples for ExiQ (**C**), HaBM (**D**), ExoS (**E**), UC (**F**), qEV (**G**) and PiCo (**H**). ND—not detectable.

For the evaluation of the variations in the different small EV purification methods, EVs obtained from each isolation approach were examined regarding particle size, particle count, and zeta potential using nanoparticle tracking analysis (NTA) via the ZetaView device. First, the particles were measured in scatter mode to obtain the size and the particle count. Second, the zeta potential measurement was performed. Overall, the EV diameters were roughly in the same range, ~130–150 nm (Figure 3A,C–H). For all purification kits, a size difference between DN/RNase-treated and untreated particles was observed. Except for ExiQ, the mean diameter was always smaller in the untreated samples. Treated particles obtained with ExiQ had an average size of only 137 nm, while DNase/RNase treatment led to particle sizes of 150–155 nm in most other kits (HaBM, ExoS, qEV and PiCo). Overall, the smallest particle sizes with a mean size of 130 nm were observed in both treated and untreated samples isolated by UC.

With respect to concentration of EVs (Figure 3B), extremely low amounts were observed for the qEV isolation method, yielding an average of 0.6 × 10^9^ particle/mL. Low EV counts were also detected with HaBM (2.4 × 10^9^ particle /mL) and UC (1.6 × 10^9^ particle/mL). Middle-range concentrations were reached using the kits of ExoS with 4.2 × 10^9^ particle/mL and PiCo with 4.9 × 10^9^ particle/mL. Interestingly, for ExoS and PiCo, the particle numbers were increased in the untreated sample fraction in comparison to DN/RNase-treated samples. By far the highest number of particles was achieved by applying the ExiQ method in combination with DNase/RNAse digestion which resulted in at least twice as many particles (9.0 × 10^9^ /mL) compared to all other kits.

The Zeta potential was also determined for all samples of all EV isolation methods (Supplementary Figure S1). The zeta potential ranged from −100 to −112 mV across all samples. Additionally, the zeta potential was in general found to be lower for the treated EVs in contrast to the untreated particles, with the exception of the ExoS isolation approach, where a higher Zeta potential was found in treated EVs.

### 2.3. Protein-Based EV Characterization

**Figure 4:**
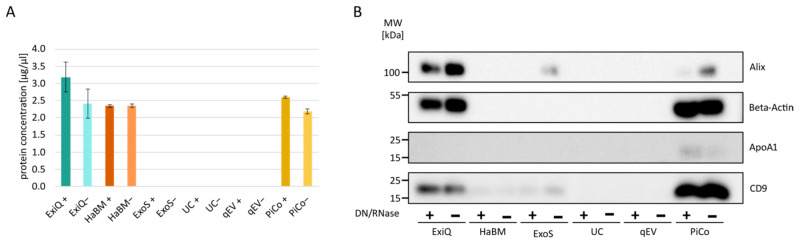
Characterization of EV proteins via Western blot. Protein concentration in µg/µL was first determined by BCA assay and depicted in a bar graph where triplicate data (*n* = 3) are presented as mean ± standard deviation (**A**). For ExoS, UC and qEV, protein content did not reach the detection range. EV specificity was checked by Western blot (**B**), showing EV-specific protein Alix and CD9, and non-EV-specific protein ApoA1. Beta-actin was used as a protein loading control.

As recommended in the MISEV 2018 and 2023 guidelines, a selection of certain proteins was analyzed via Western blot to demonstrate the presence of extracellular vesicles and assess their degree of purity after harvest using different isolation methods. In detail, CD9 was chosen as an EV-transmembrane protein, Alix as a cytosolic protein recovered in EVs, and ApoA1 as a representative for non-EV co-isolated structures (lipoprotein). Before carrying out the Western blot, protein concentration was determined via BCA assay (Figure 4A). For ExoS, UC and qEV isolations, the obtained protein amounts were below the lower detection limit of the BCA assay. The protein concentration for HaBM and PiCo isolation was in the range of 2.2 and 2.6 µg/µL, with little difference between DNase/RNase-treated or untreated EV isolates. The highest protein concentration was measured in EV preparations derived from the ExoQ kit. For the DN/RNase-treated ExoQ samples, the concentration was 3.2 µg/µL, whereas untreated EV samples displayed 2.4 µg/µL protein.

On the Western blot (Figure 4B), selected markers were not visible for UC and qEV isolations, because the protein concentration, as mentioned before, was too low to adjust for 30 µg/lane protein input. For all other EV isolation methods, tetraspanine-9 (CD9), an EV-specific transmembrane protein of 22 kDa, was visible and showed the strongest intensity for ExiQ and PiCo kits (Supplementary Figure S2). Further evidence for EV-specific protein expression came from Alix, a cytosolic protein of 95 kDa found in EVs, which was detectable in samples derived from ExiQ, ExoS and PiCo isolations but not in HaBM. Here, ExiQ also had the strongest signal for this protein. Beta-actin, a cytoskeletal protein of 42 kDa used as a protein loading control, was only detected in ExiQ and Pico isolations. Finally, the purity of isolated EVs was determined by testing for 25 kDa ApoA1, an apolipoprotein used as evidence of co-isolated non-EV structure and lipoproteins, respectively, which was only observed in traces for PiCo isolations. In contrast, ExiQ did not show any ApoA1 signal resulting in EVs, not displaying any lipoprotein coprecipitation.

### 2.4. Comparison of Different EV Extraction Methods with Respect to Obtained DNA Yields

**Figure 5:**
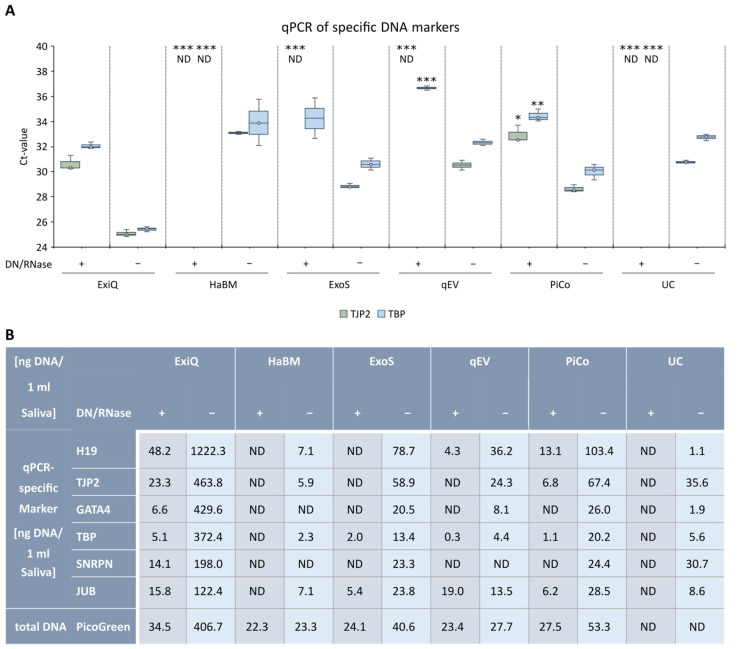
Comparison of EV isolation kits with respect to DNA yields. (**A**) qPCR expression results of TJP2 and TBP are highlighted, and obtained Ct-values are shown as boxplots for the following EV extraction kits: miRCURY exosome serum plasma (ExiQ), Immunobeads for overall exosome capture (HaBM), Exo-spin (ExoS), qEVoriginal (qEV), Pierce concentrator (PiCo) and ultracentrifugation (UC). A t-test was performed comparing the Ct-values of enzyme-treated ExiQ-isolated EVs with treated EVs from all other isolation approaches. Obtained *p*-values (*p* > 0.05 *, *p* > 0.01 **, *p* > 0.001 ***) are displayed. (**B**) Calculated mean DNA amounts (ng) obtained from 1 mL of cell-free saliva are summarized in a table. In detail, the mean quantities of the 6 tested specific qPCR markers (ng DNA/1 mL saliva) are shown in comparison with the total DNA amount measured via PicoGreen. For each kit, values resulting from DNase/RNase-treated EVs (+) are shown in contrast with untreated EVs (−). Tests with no measurable DNA quantities are labeled as “ND” (“not detectable”).

To evaluate EV DNA yield obtained from the different EV isolation kits, DNA quantities were spectroscopically measured and qPCR on six selected gene markers (H19, TJP2, GATA4, TBP, SNRPN and JUB) (Supplementary Table S1) was performed. DNA extraction efficiency of every tested EV kit was checked by comparing Ct-values and calculating ng quantities of EV DNA obtained from 1 mL of cell-free saliva. As an example, the results of the gene markers TLP2 and TBP are illustrated in Figure 5A as boxplots. The total DNA concentrations obtained for each specific qPCR marker, in contrast to the total amount of isolated DNA, are compiled as a table in Figure 5B. The digestedCt–undigestedCt differences, displaying higher Ct-values for DN/RNase digested samples, varied for each kit, ranging from four Ct-values difference for the PiCo kit to a maximum of six Cts difference for the ExiQ approach. For the kits HaBM, ExoS, qEV and UC, this ratio could not be calculated since digested samples were not detectable in the qPCR. When measuring total DNA quantities via PicoGreen fluorescence spectroscopy, the same differences between DNase1/RNaseA-treated and untreated samples were observed as in qPCR. These observed differences between treated and untreated samples are a clear indication that a significant amount of DNA is obviously attached to the EV surface. Comparing the EV isolation methods with detectable DNA concentrations after nuclease treatment, the best yield was obtained with the miRCURY Exosome Isolation kit—serum and plasma (ExiQ).

### 2.5. Comparison of Different EV Extraction Methods with Respect to Obtained RNA Yields

**Figure 6:**
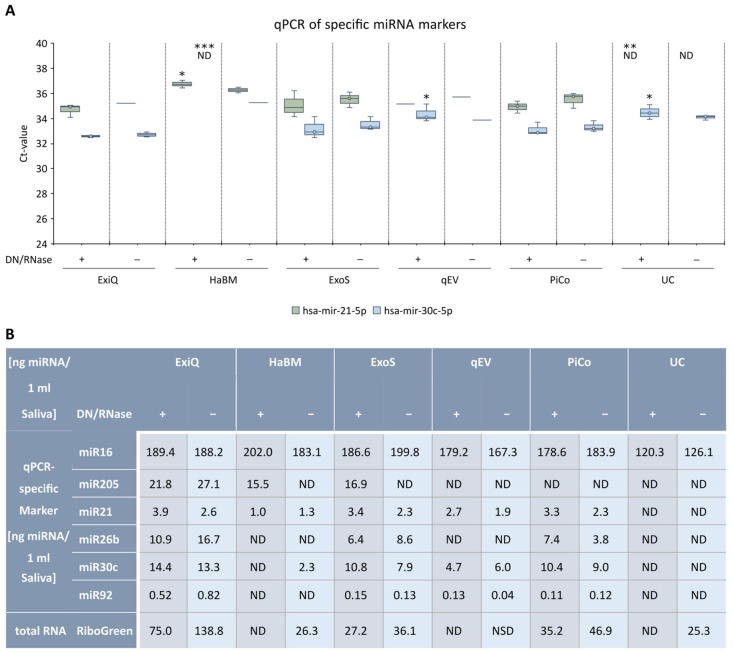
Comparison of EV isolation kits with respect to miRNA yields. (**A**) qPCR expression results of hsa-mir-21-5p and hsa-mir-30c-5p are highlighted and shown as boxplots of Ct-values obtained for the following EV extraction kits: miRCURY exosome serum plasma (ExiQ), Immunobeads for overall exosome capture (HaBM), Exp-spin (ExoS), qEVoriginal (qEV), Pierce concentrator (PiCo) and ultracentrifugation (UC). A t-test was performed comparing the Ct-values of enzyme-treated ExiQ-isolated EVs with treated EVs from all other isolation approaches. Obtained *p*-values (*p* > 0.05 *, *p* > 0.01 **, *p* > 0.001 ***) are displayed. (**B**) Calculated mean RNA amounts (ng) RNA, miRNA, respectively, obtained from 1 mL of cell-free saliva are summarized in a table. In detail, the quantities of the 6 tested miRNA markers (ng miRNA/1 mL saliva) are shown in comparison with the total RNA amount. For each kit, values resulting from DNase 1/RNase A-treated EVs (+) are shown in contrast with untreated EVs (−). Tests with not-measurable RNA/miRNA quantities are labeled as “ND” (“not detectable”).

Besides looking at DNA yields, we evaluated the six given EV preparation kits also for their feasibility to extract EV miRNA from cell-free saliva. For the kit comparison, an equal volume of RNA eluate after exosomal RNA isolation was put into each qPCR reaction of a set of six tested miRNA markers (hsa-mir-16-5p, hsa-mir-205-5p, hsa-mir-21-5p, hsa-mir-26b-5p, hsa-mir-30c-5p and hsa-mir-92-5p) (Supplementary Table S2). We evaluated the miRNA extraction efficiency by comparing Ct-values and calculated miRNA ng quantities obtained from 1 mL of cell-free saliva. As an example, the results of marker hsa-mir-21-5p and hsa-mir-30c-5p are shown as boxplots in Figure 6A. The total miRNA concentrations received for each specific miRNA marker, in contrast to the total RNA amount measured spectroscopically, are compiled in a table in Figure 6B. We again calculated the digestedCt–undigestedCt differences for each EV kit, but this time, hardly any difference in nuclease-treated and untreated samples could be observed, indicating that, in contrast to DNA, no significant amount of RNA is attached outside of EVs. Total RNA quantities obtained from DNase1/RNaseA-treated and untreated samples were measured via RiboGreen fluorescence dye. Comparing the EV preparation methods with detectable miRNA concentration after nuclease treatment, the best yield was again obtained using the miRCURY Exosome Isolation kit (ExiQ).

## 3. Discussion

With the increasing number of studies focusing on EVs, the ISEV board established guidelines to define a minimal set of biochemical, biophysical and functional standards for the field [30]. These standards, first introduced in 2014 and later updated in the MISEV2018 [3] and MISEV2023 [4] guidelines, aim to promote consistency and rigor in EV research. Nevertheless, even in its most recent version, the guideline authors admit unresolved challenges, particularly concerning the identification of specific markers for EV subtypes and the lack of standardized, contamination-free EV preparation and proper EV characterization [4]. EVs hold great potential for clinical diagnostics, as they carry a range of bioactive cargo molecules, originate from diverse tissues/cells, including diseased ones, and circulate in easily accessible body fluids. This potential for use in clinical diagnostics implies the need for the development of reliable, standardized EV isolation methods.

The primary objective of our study was a comprehensive comparison of six commercially available EV isolation methods for their suitability in processing cell-free saliva. We aimed to identify the most effective protocol for potential clinical applications. Saliva is a tremendously attractive source for EVs due to its completely non-invasive, simple and pain-free collection. Despite these advantages, saliva remains relatively underexplored, and most available kits are optimized for the application in serum, plasma and cell culture media rather than saliva.

**Figure 7:**
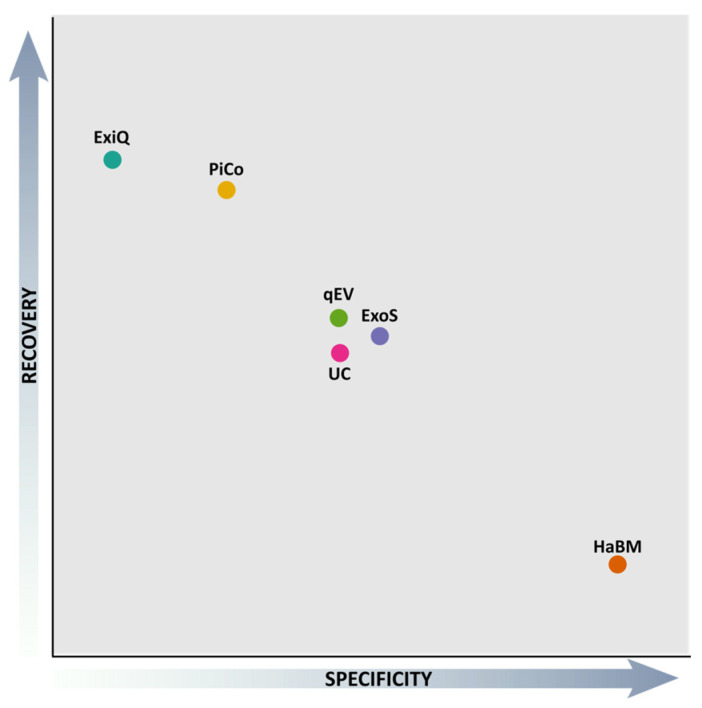
Technique classification. The tested commercially available isolation methods were ranked in a grid regarding the recovery and specificity of extracted salivary extracellular vesicles.

In this study, the six tested methods encompass a wide range of EV isolation principles and ranged from very specific techniques, such as immuno-capturing beads and size-exclusion chromatography, to more general and simpler approaches, such as polymer-based precipitation and ultrafiltration. All salivary isolation methods applied in this study were classified based on their recovery and specificity, as illustrated in Figure 7, following a format previously developed by Théry et al. [31].

Each isolation method was thoroughly evaluated based on vesicle size, concentration, purity and EV cargo content.

Regarding the size, all protocols successfully recovered vesicles within the expected size range of 130–150 nm, confirming their ability to isolate small EVs. The applied DNase/RNase treatment resulted in a shift in the diameter of the vesicles, suggesting that a significant fraction of molecules is attached to the EV surface [32,33,34]. However, the decrease in DNA cannot be attributed exclusively to EV surface-associated DNA, as co-isolated cell-free DNA, protein–DNA complexes, residual cellular debris, bacterial DNA, or DNA associated with non-EV nanoparticles may also contribute to the observed effect [35]. The EV yields varied strongly with each extraction technique. The ExiQ kit produced more than twice the number of vesicles compared to the other kits, whereas the HaBM, qEV and UC kits generated the lowest vesicle yields, followed by the ExoS and Pico extraction approaches. This variation appears to reflect a trade-off between specificity and recovery; more specific methods typically yielded fewer vesicles.

Protein quantification was below detection thresholds in three out of the six isolation methods, aligning with their lower vesicle yields. Ultrafiltration was found not to be a proper EV isolation technique, as ApoA1 detection indicated co-isolation of lipoproteins. Although ExoS showed some EV-specific markers, their expression was weak. The ExiQ kit demonstrated the highest protein yield with the presence of EV-specific proteins and the absence of contaminant markers, indicating decent EV purity.

To assess nucleic acid cargo, we analyzed DNA and miRNA levels in both nuclease-treated and -untreated samples. DNA levels were significantly reduced after nuclease treatment, confirming the presence of surface-bound DNA. No such effect was observed for miRNA, which therefore appears to be only encapsulated within vesicles. Among all tested methods, the miRCURY Exosome Isolation kit—serum and plasma (ExiQ) produced the highest yields of both DNA and miRNA and achieved the best results in all evaluation categories, as illustrated in Table 1.

To the best of our knowledge, our work represents the up-to-date most extensive evaluation of salivary EV isolation techniques. While numerous studies have focused on purification of EVs from urine, serum, plasma, and cell culture media [18,19,33,36,37,38], only a few have offered such a comprehensive comparison of techniques—even in those more commonly studied fluids. Notably, there is currently no equally systematic and in-depth study available for saliva. Our work therefore fills a critical gap, providing a broad, side-by-side assessment of multiple commercially available EV isolation methods applied specifically to saliva. There are only two studies of similar comprehensiveness on serum-derived EVs [17,36]. A few smaller-scale studies have addressed salivary EVs [37,39,40,41], but none of them offer the same spectrum of methodological comparison as presented here.

Among all tested methods, the miRCURY Exosome Isolation kit—serum and plasma (ExiQ) performed best across all evaluation categories. It offers a standardized user-friendly workflow and requires only a benchtop centrifuge, making it particularly well-suited for clinical routine use. For this fast and straightforward approach, Western blot analysis confirmed both high EV purity and the absence of major contaminants. Although precipitation-based methods can carry a risk of lipoprotein co-isolation [42], this issue was not prominent in our study using the ExiQ kit. Nevertheless, the protocol includes manual steps that may introduce variability; thus, precision and careful execution remain critical. One notable advantage is the flexibility of the final EV suspension, which can be adapted to any volume and buffer for downstream applications such as real-time qPCR, RNA sequencing, or DNA methylation profiling. The miRCURY Exosome Isolation—serum and plasma kit (ExiQ) approach comes with a standardized protocol along with the commercially distributed kit, which should enable reproducible and consistent isolations, thereby ensuring comparability of studies [43].

The compatibility of the ExiQ isolated with downstream analysis was demonstrated through the successful detection of both DNA and miRNA via qPCR. Further application of this method is underway using clinical samples, with plans to conduct high-throughput omics studies such as DNA methylation profiling using bead microarrays and small RNA sequencing.

## 4. Materials and Methods

### 4.1. Sample Collection and Further Processing

Saliva samples were donated by healthy volunteers (all working at the Austrian Institute of Technology) and judged by the local ethics committee of the city of Vienna not to require any ethical approval, as the intended use comprised molecular method evaluation and optimization. Whole saliva was collected from 4 healthy individuals (2 males, 2 females) by unstimulated spitting into a sterile 50 mL Falcon tube. Saliva donors were not allowed to smoke, eat or drink one hour before saliva donation. Ten minutes before starting collection, each donor had to rinse his/her mouth with water without swallowing it. After a minimum of 15 mL saliva per donor was collected, samples were centrifuged at 3000× *g* at 4 °C for 20 min, and cell-free supernatant from each sample was carefully transferred and pooled into a sterile glass bottle without interrupting the cell pellet. Aliquots of 1 mL from the pooled cell-free saliva were stored at −80 °C.

### 4.2. EV Isolation from Saliva

For the comparison of various EV extraction methods, six commercially available kits were used (Table 2): miRCURY Exosome Isolation kit—Serum and Plasma (ExiQ) (76603, Qiagen, former Exiqon, Hilden, Germany); immunobeads for overall exosome capture from human biological fluids—plasma, serum, urine (HaBM) (HBM-BOLF-C/10-04, HansaBioMed, Tallinn, Estonia); Exo-Spin Exosome Purification Kit for cell culture media/urine/saliva (ExoS) (EX01, Cell guidance systems, Cambridge, UK); qEVoriginal Size Exclusion Column (qEV) (qEVori, iZON, Oxford, UK); Pierce Concentrator, PES, 100K MWCO, 0.5 mL (PiCo) (88503, Thermo Fisher Scientific, Waltham, MA, USA) and differential ultracentrifugation (UC). The starting input volume for each EV isolation was 1 mL of cell-free saliva derived from a pool of cell-free saliva. Six EV isolations per kit type were done from 1 mL of saliva each according to the manufacturer’s instructions, including potential adjustment related to the saliva input volume and minor modifications. Isolated from 1 mL cell-free saliva-derived EVs were finally resuspended in 300 µL 1× PBS each. In the following step, 3 out of the 6 EV isolation replicates per kit were treated with DNase 1 and RNase A (Section 4.3). Thus, each isolation method was tested using untreated EV samples in triplicate and treated EV samples in triplicate.

### 4.3. EV Treatment Using DNase and RNase

The isolated EVs resuspended in 300 µL 1× PBS were treated with 8 µL DNase 1, RNase-free (1 U/µL) (EN0521, Thermo Fisher Scientific, Waltham, MA, USA) and 3 µL of RNase A, DNase and Protease-free (10 µg/µL) (EN0531, Thermo Fisher Scientific, Waltham, MA, USA). For the activity of the DNase 1, 30 µL of the 10× reaction buffer with MgCl_2_ (B43, Thermo Fisher Scientific, Waltham, MA, USA) was also added to the reaction. The reaction was incubated for 1 h at 37 °C with slight shaking at 300 rpm.

### 4.4. DNA and RNA Isolation from Salivary EVs

The DNA and RNA extraction from the salivary EVs was performed according to the manufacturer’s instructions of ZR-Duet DNA/RNA MiniPrep kit (D7001, Zymo Research, Irvine, CA, USA), which allows simultaneous DNA and RNA isolation in separate fractions. The starting sample volume of 341 µL resuspended EVs (resulting from EV isolation of 1 mL cell-free saliva and after DNase/RNase digest) was mixed with 3 volumes of DNA/RNA Lysis Buffer, followed by transferring the whole volume to a Zymo-Spin IIIC column. All subsequent steps were performed according to the manufacturer’s protocol. Both DNA and RNA were eluted in 100 µL DNase/RNase-free water each. The isolated DNA was stored at −20 °C and the RNA at −80 °C until further analysis.

### 4.5. Quantification of EV-Derived DNA and RNA

The total amount of DNA obtained from EVs isolated via six different EV approaches was determined using the Quant-iT PicoGreen dsDNA kit (P11496, Thermo Fisher Scientific, Waltham, MA, USA) and NanoDrop 3300 (Thermo Fisher Scientific, Waltham, MA, USA) following the producer’s manual.

For the determination of the total RNA concentrations achieved from the various EV isolation kits, the Quant-iT RiboGreen RNA kit (R11490, Thermo Fisher Scientific, Waltham, MA, USA) was used.

### 4.6. Real-Time Quantitative PCR (qPCR) for Selected DNA Targets

DNA amounts obtained from the different EV isolation approaches were determined via qPCR by testing six specific control genes for which qPCR assays had been established in our lab before (Supplementary Table S1). An aliquot of each DNA eluate corresponding to 20 µL of the initial cell-free saliva sample was tested per qPCR reaction. The single qPCR reaction contained 1× PCR buffer with MgCl_2_ (part of 203203, Qiagen, Hilden, Germany), 0.16 mM of each dNTP (R0181, Thermo Fisher Scientific, Waltham, MA, USA), 0.28× EvaGreen dye (31000, Biotium, Fremont, QC, Canada), 0.03 Units HotStarTaq DNA Polymerase (203203, Qiagen, Hilden, Germany), 0.2 µM of each forward and reverse primer (Supplementary Table S1), 2 µL of the 100 µL DNA isolation eluate and was filled up to the total volume of 12 µL with nuclease-free water. All qPCR reactions were performed in a 384-well format on a LightCycler 480 instrument (Roche, Basel, Switzerland) under the following cycling conditions: 15 min at 95 °C and 45 cycles of 40 s at 95 °C, 40 s at 65 °C, and 80 s at 72 °C. A final step of the amplification reaction included 7 min at 72 °C. Accuracy of Ct-values was monitored via melting curve analysis of each resulting PCR product. A calibration curve from a pool of purified DNA from whole blood was used for the calculation of absolute DNA amounts.

### 4.7. Multiplex Stem-Loop Real-Time Quantitative PCR (qPCR) for Selected microRNA Targets

MicroRNA amounts obtained after RNA isolation from EVs provided by the 6 different approaches were determined via qPCR for six specific microRNA species. MicroRNA targets were selected based on the fact that the assays had already been established before in our lab, applying the stem-loop RT-PCR method [44]. Reverse transcription (RT) reaction was performed using the TaqMan MicroRNA Reverse Transcription kit (4366596, Thermo Fisher Scientific, Waltham, MA, USA). An aliquot of each RNA eluate corresponding to 50 µL of the initial 1 mL of cell-free saliva sample was added per RT reaction. The single RT reaction contained 1x Reverse Transcription buffer, 1 mM of each dNTP, 0.25 Units RNase inhibitor, 3.33 Units MultiScribe reverse transcriptase, 2 nM of RT-loop primer, 5 µL of the 100 µL RNA isolation eluate, and was filled up to the total volume of 15 µL with nuclease-free water. The following cycling parameters were used for the RT reaction: 30 min at 16 °C; 60 cycles of 30 s at 20 °C, 30 s at 42 °C, 1 s at 50 °C; and finally 5 min at 85 °C. The resulting cDNA product was diluted 1:3 with nuclease-free water. The subsequent single qPCR reaction contained 1xPCR buffer with MgCl_2_ (part of 203203, Qiagen, Hilden, Germany), 0.16 mM of each dNTP (R0181, Thermo Fisher Scientific, Waltham, MA, USA), 0.28× EvaGreen dye (31000, Biotium, Fremont, QC, Canada), 0.03 Units HotStarTaq DNA Polymerase (203203, Qiagen, Hilden, Germany), 1.5 µM of forward primer, 0.7 µM of universal reverse primer (Supplementary Table S2), and 2 µL of the diluted cDNA sample and was filled up to the total volume of 12 µL with nuclease-free water. All qPCR reactions were performed under the following cycling conditions: 5 min at 95 °C; followed by 45 cycles of 40 s at 95 °C, 40 s at 60 °C, 80 s at 72 °C; and finally 7 min at 72 °C. Accuracy of Ct-values was monitored via melting curve analysis of each resulting PCR product. A calibration curve from the universal human miRNA reference (750700, Agilent, Santa Clara, CA, USA) was used for the calculation of absolute miRNA amounts.

### 4.8. Nanoparticle Tracking Analysis (NTA)

The extracted extracellular vesicles were quantified via the Brownian motion of particles using ZetaView nanoparticle tracking video microscope (PMS-120, Particle Metrix, Meersbusch, Germany). Extracted EV sample aliquots were diluted 50- to 500-fold with 1× PBS to achieve an optimal concentration of a minimum of 10^6^ particle/mL for NTA. For each measurement, 1 mL of diluted sample was injected into the measurement chamber. Vesicle size and concentration were determined by visualization of the particles’ Brownian motion, applying laser light scattering, and tracking over time. Subsequently, the zeta potential was determined by the electrophoretic motion of the particles, depending on the speed due to electrostatic attraction in the electric field. All measurements were performed with pre-acquisition parameters set to sensitivity of 75, shutter of 50, frame rate of 30, and temperature at 24 °C. The post-acquisition was adjusted to a brightness of 20. Polystyrene beads with a known size of 100 nm (110-0020, Particle Metrix) were used to determine the accuracy of the instrument during daily instrument preparation. The data were analyzed via ZetaView device software (8.04.02).

### 4.9. EV Characterization via Western Blot

For lysis, 5 parts of EV sample were mixed with 1 part of 5× RIPA buffer (Tris-HCl pH 7.4, 0.25 M; NaCl 0.75 M; EDTA, 5 mM; Triton X-100, 5%; Sodium deoxycholate, 0.05%) plus PhosSTOP (04906837001, Basel, Switzerland) and cOmplete ULTRA tablets, mini (05892970001, Basel, Switzerland) which were incubated for 20 min on ice. The protein concentration of the lysed EV solution was determined using the Pierce BCA protein assay kit (23227, Thermo Fisher Scientific, Waltham, MA, USA). For Western blot analysis, 30 µg of EV proteins were prepared with Laemmli buffer, incubated for 5 min at 95 °C, placed on ice, and immediately after, finally transferred to the gel slots for running on 10% Tris-Glycine SDS-PAGE at 120 V. The proteins were blotted onto an Immune-blot PVDF membrane (1620177, Bio-Rad Laboratories, Hercules, CA, USA) at 40 mV overnight at 4 °C. The membrane was blocked with 5% non-fat dried milk powder (A0830, AppliChem, Darmstadt, Germany) in PBST for 2 h at room temperature. The membrane was incubated with anti-Alix mouse antibody (3A9, 1:1000) (2171S, cell signaling technology, Danvers, MA, USA), anti-CD9 rabbit antibody (D3H4P, 1:1000) (13403S, cell signaling technology, Danvers, MA, USA), anti-ApoA1 mouse antibody (5F4, 1:1000) (3350S, cell signaling technology, Danvers, MA, USA) and anti-ß-actin-POX antibody (1:20,000) (A3854, Sigma Aldrich, St. Louis, MO, USA) while shaking overnight in a cold room. After washing with PBST buffer for 30 min, the membrane was then incubated with anti-mouse IgG HRP-linked horse antibody (1:5000) (7076, Cell Signaling Technology, Danvers, MA, USA) and anti-rabbit IgG HRP-linked sheep antibody (1:5000) (NA934, Cytiva Life Science, Marlborough, MA, USA) for 1 h at room temperature. Finally, the protein bands were visualized using the Clarity Western ECL substrate kit (170-5060, Bio-Rad Laboratories, Hercules, CA, USA) according to the manufacturer’s manual.

## Figures and Tables

**Table 1:** Overview of the results in various evaluation categories comparing all tested EV extraction techniques. All results were evaluated subjectively. The red colored dots indicate the absence of data. The yellow dots denote worse or lower values in comparison to the best performing approach, while green dots represent the best result.

	ExiQ	HaBM	ExoS	qEV	PiCo	UC
EV size	●	●	●	●	●	●
EV amount	●	●	●	●	●	●
Western Blot	●	●	●	●	●	●
DNA	●	●	●	●	●	●
miRNA	●	●	●	●	●	●
Protein	●	●	●	●	●	●

**Table 2:** Overview of tested EV isolation kits and methods.

Abbreviation	Kit Name	Manufacturer	Established For	Preparation Principle
ExiQ	miRCURY Exosome Serum/plasma kit	Qiagen (Hilden, North Rhine-Westphalia, Germany)	Serum, Plasma	Precipitation
HaBM	Immunobeads for Overall Exosome capture	Hansa Bio Med (Tallinn, Harju County, Estonia)	Serum, Plasma, Urine, cell culture media	Immuno-beads
ExoS	Exo-spin™ kit	Cell Guidance Systems (Cambrdige, UK)	Urine, cell culture media, Saliva	Size-exclusion chromatography
UC	Ultracentrifugation	-	Cell culture media	Differential centrifugation
qEV	qEVoriginal	iZON (Christchurch, Canterbury, New Zealand)	Serum, Plasma, Urine, cell culture media, Saliva	Size-exclusion chromatography
PiCo	Pierce Concentrator, PES, 100K MWCO	Thermo Fisher Scientific (Waltham, MA, USA)	Biological samples	Size-exclusion

## Data Availability

The original contributions presented in this study are included in the article/Supplementary Materials. Further inquiries can be directed to the corresponding author.

## Supplementary Materials

The following supporting information can be downloaded at: https://www.mdpi.com/article/10.3390/ijms27114899/s1.

## Abbreviations

The following abbreviations are used in this manuscript:

EV	Extracellular vesicle
ILVs	Intraluminal vesicles
miRNA	microRNA
MISEV	Minimal information for studies of extracellular vesicles
MVEs	Multivesicular endosomes
NTA	Nanoparticle tracking analysis
PBS	Phosphate-buffered saline
PBST	Phosphate-buffered saline with Tween ^®^ detergent
qPCR	Quantitative PCR
RT	Reverse transcription
UC	Ultracentrifugation
