# Supplementary material for: Comparison of Methods for the Isolation of Salivary Extracellular Vesicles
Source: Int J Mol Sci. 2026 May 28;27(11):4899. doi: 10.3390/ijms27114899 (PMC13257335; doi:10.3390/ijms27114899)
Supplement: Supplementary file 1 [file ijms-27-04899-s001.zip › ijms-4300203-supplementary.pdf]

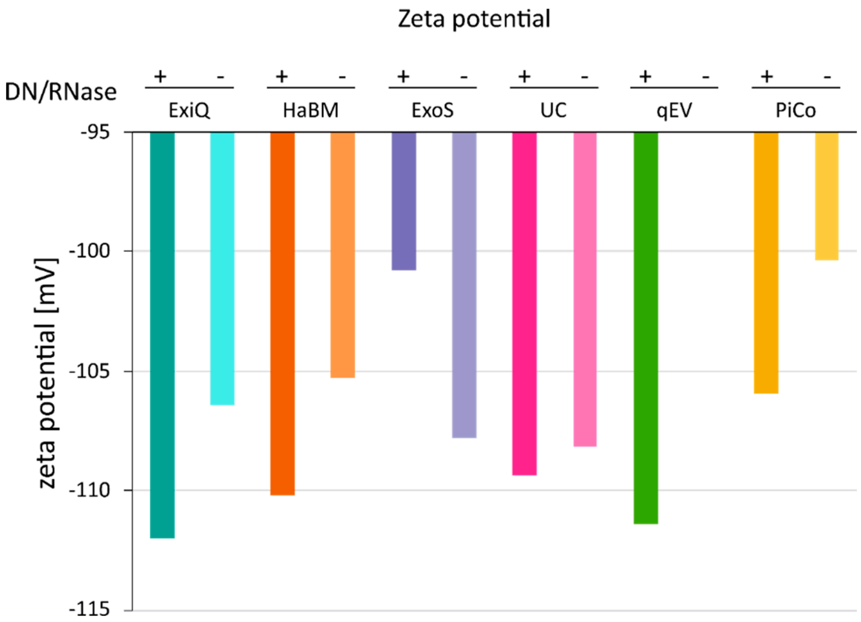


**Figure S1.** Zeta potential.

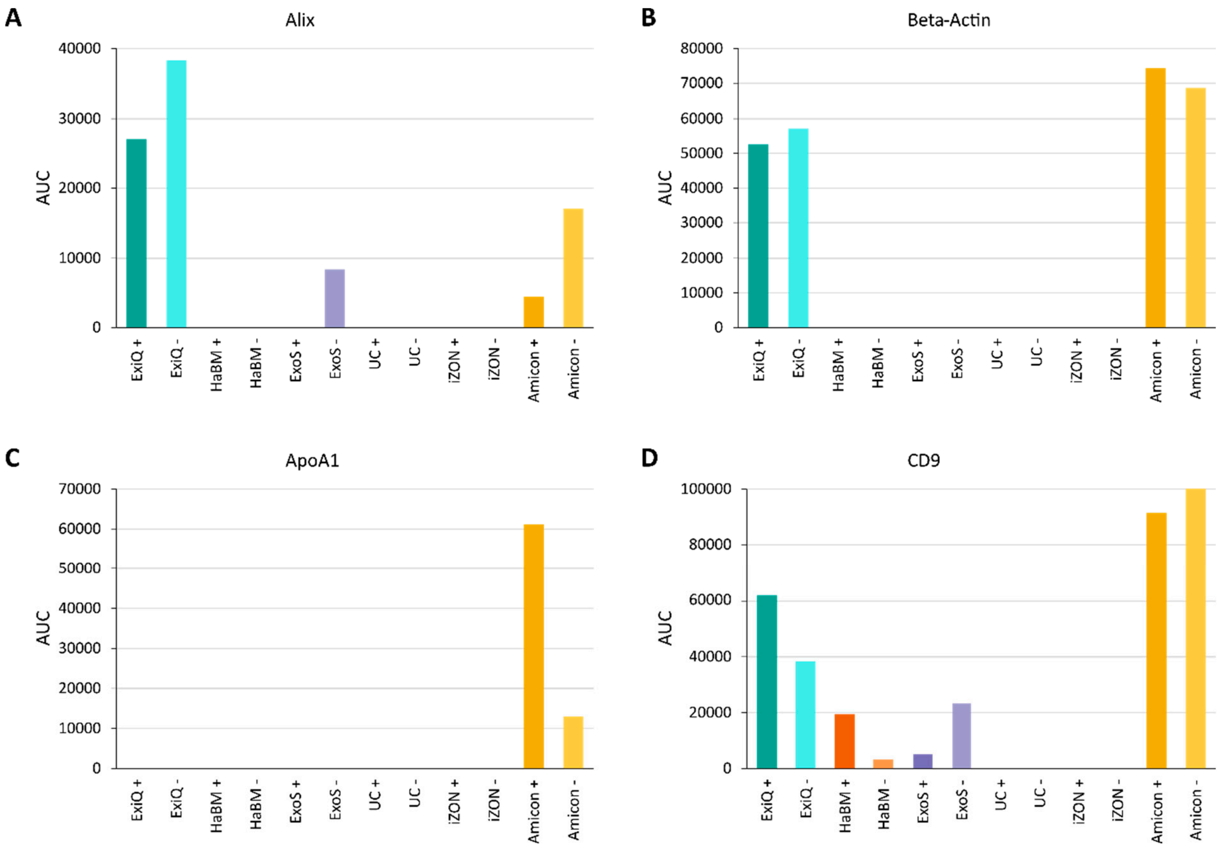


**Figure S2.** Densitometry of Western blot.

| Gene | Forward primer sequence | Reverse primer sequence | Product length |
|---|---|---|---|
| H19 | TGG GCC GCA GTG CCT CGT GGG | GGG CGA AGC GGC CAC GGG AG | 101 |
| SNRPN | GTC CCC CAT CCG CCC CCA ACT G | CCC ACT GCG GTT ACC CCG CAT GCT C | 105 |
| JUB | GGC ATT GCT CTG CCC ATA GAT GCC TTT G | GGA ATC CCT GGT TTT GAC CTG GGG GA | 101 |
| GATA4 | GCC GGG GTC GCG GAC TGC CA | CGC GCT GCC CCA GGG ATT CCA | 105 |
| TBP | GGC CCG CGG CTC TGT GCG | GTG TCG GAT CCG CAG GCG CAG | 105 |
| TJP2 | TGT GCC GCG CGG TTG GGA GG | CAG CTT CCT ACG GCG CAT CGG GGA | 106 |

**Table S1.** Forward and reverse primer sequences of qPCR DNA targets.

| miRNA | Forward primer sequence | Universal reverse primer sequence |
|---|---|---|
| miR-16 | CGC GCT AGC AGC ACG TAA AT | GTG CAG GGT CCG AGG T |
| miR-21 | GCC CGC TAG CTT ATC AGA CTG ATG | GTG CAG GGT CCG AGG T |
| miR-30c-5p | AGC CGC CTG TAA ACA TCC TAC ACT | GTG CAG GGT CCG AGG T |
| miR-205 | CGC TCC TTC ATT CCA CCG G | GTG CAG GGT CCG AGG T |
| miR-26b | CGC CGC TTC AAG TAA TTC AGG AT | GTG CAG GGT CCG AGG T |
| miR-92 | GCC TGT ATT GCA CTT GTC CCC | GTG CAG GGT CCG AGG T |

**Table S2.** List of tested miRNA targets including forward and universal reverse primer sequences.
